# Population Structure of the South West Indian Ocean Islands: Implications for Precision Medicine

**DOI:** 10.3389/fgene.2021.758563

**Published:** 2021-11-23

**Authors:** Anisah W. Ghoorah, Toto Chaplain, Rakotoarivony Rindra, Smita Goorah, Ganessen Chinien, Yasmina Jaufeerally-Fakim

**Affiliations:** ^1^ University of Mauritius, Reduit, Mauritius; ^2^ University of Toamasina, Barikadimy, Toamasina, Madagascar; ^3^ University of Antananarivo, Antananarivo, Madagascar

**Keywords:** precision medicine, genetics, South West Indian Ocean, demography, population structure, disease variants

## Abstract

Precision medicine has brought new hopes for patients around the world with the applications of novel technologies for understanding genetics of complex diseases and their translation into clinical services. Such applications however require a foundation of skills, knowledge and infrastructure to translate genetics for health care. The crucial element is no doubt the availability of genomics data for the target populations, which is seriously lacking for most parts of Africa. We discuss here why it is vital to prioritize genomics data for the South West Indian Ocean region where a mosaic of ethnicities co-exist. The islands of the SWIO, which comprise Madagascar, La Reunion, Mauritius, Seychelles and Comoros, have been the scene for major explorations and trade since the 17th century being on the route to Asia. This part of the world has lived through active passage of slaves from East Africa to Arabia and further. Today’s demography of the islands is a diverse mix of ancestries including European, African and Asian. The extent of admixtures has yet to be resolved. Except for a few studies in Madagascar, there is very little published data on human genetics for these countries. Isolation and small population sizes have likely resulted in reduced genetic variation and possible founder effects. There is a significant prevalence of diabetes, particularly in individuals of Indian descent, while breast and prostate cancers are on the rise. The island of La Reunion is a French overseas territory with a high standard of health care and close ties to Mauritius. Its demography is comparable to that of Mauritius but with a predominantly mixed population and a smaller proportion of people of Indian descent. On the other hand, Madagascar’s African descendants inhabit mostly the lower coastal zones of the West and South regions, while the upper highlands are occupied by peoples of mixed African-Indonesian ancestries. Historical records confirm the Austronesian contribution to the Madagascar genomes. With the rapid progress in genomic medicine, there is a growing demand for sequencing services in the clinical settings to explore the incidence of variants in candidate disease genes and other markers. Genome sequence data has become a priority in order to understand the population sub-structures and to identify specific pathogenic variants among the different groups of inhabitants on the islands. Genomic data is increasingly being used to advise families at risk and propose diagnostic screening measures to enhance the success of therapies. This paper discusses the complexity of the islands’ populations and argues for the needs for genotyping and understanding the genetic factors associated with disease risks. The benefits to patients and improvement in health services through a concerted regional effort are depicted. Some private patients are having recourse to external facilities for molecular profiling with no return of data for research. Evidence of disease variants through sequencing represents a valuable source of medical data that can guide policy decisions at the national level. There are presently no such records for future implementation of strategies for genomic medicine.

## Introduction

The SWIO covers four island states off the shores of Eastern and Southern Africa with the largest one being Madagascar; the others include Mauritius, Seychelles, Comoros and a French island territory (La Reunion). Geographical proximity has contributed to all the islands having similar historical paths and were at times considered as a single entity during the colonial period. This region has been an important trading route between Europe and Asia since the 18th century. There were many events of migration for various reasons but mainly as importation of labor from mainland Africa and India. This activity has shaped the present day demography of the region resulting in a unique diversity and cultural traditions.

All the islands share some common history of sugarcane plantations, the use of both French and English language and the Creole dialects of French origin. The demographics have been shaped through colonization by the Dutch, British and French since the 17th century, by the slave trade in the 18th and 19th centuries and by the arrival of laborers and traders from India. According to Gervese Clarence-Smith (1989) more than 200,000 slaves were brought to sugarcane plantations from the coasts of East Africa and Madagascar. The population structure of this part of Africa is unique in its blend of genetics, lifestyle and environment which calls for an in-depth investigation drive innovation in the health sector. The demography of Mauritius reflects the origin of its settlers who were mostly Indian, African, Chinese, European (Table.1). This has led to an important admixed section of the population. Rodrigues, an island part of the Republic of Mauritius, has a higher proportion of African ancestry. Seychelles consists of 115 islands scattered over 1.3 m sq km of ocean. With a population of just over 96,000. with ethnicities being mostly African, and some European, Chinese and Indian. The Comoros consist of three islands with a total size of 1,660 sq km lying between the East African coast and Madagascar and has mostly Afro-Arab and Malagasy groups. Madagascar has a population of over 27 m with ethnicities from mostly a mix of Austronesian and African and to a lesser extent European and Indian. The presence of Arabs is noted from the 9th to the 15th century on the North-West and oriental coast of Madagascar (Vérin, 1975). First, the Indonesian migrants reached residents of the Indian Ocean through East of Africa, where they would have been in contact with other migrants including the Bantus (Deschamps, 1965; Boiteau, 1982). Studies have shown that at least three great African migrant waves have contributed to the Malagasy population (Rakotoarivony et al., 2019).

**TABLE 1:** Population diversity.

Country	Males^a^	Females^a^	Population (thousands)^a^	Ethnic diversity (DELF)^b^	Ethnic composition %^c^
Comoros	448	440	888	0.041 (rank 192 on 210)	97 Comorian
					1.6 Makua
Madagascar	14,184	14,244	28,427	0.255 (rank 94 on 210)	34.3 Malagasy
					24 Merina Malagasy
					13.4 Betsimisaraka Malagasy
					11.3 Betsileo Malagasy
					7.0 Tsimihety Malagasy
					5.9 Sakalava Malagasy
					1.1 Makua
					Rest French/Other
Mauritius^d^	628	646	1,273	0.560 (rank 6 on 210)	67 South Asian
					27.4 Mixed
					3 Chinese
					2.6 Other (eg European)
Reunion	436	465	902	NA	25.6 European
					23 South Asian
					3.4 Chinese
					42.6 Mixed
Seychelles	51	48	99	0.070 (rank 184 on 210)	91.4 Seychelles
					4.4 South Asian

a United Nations, Department of Economic and Social Affairs, Population Division (2019). World Population Prospects 2019, custom data acquired via website. https://population.un.org/wpp/DataQuery/

b Kolo P (2012) “Measuring a New Aspect of Ethnicity - The Appropriate Diversity Index,” Ibero America Institute for Econ. Research (IAI) Discussion Papers 221, Ibero-America Institute for Economic Research. Definition: The DELF measures the expected dissimilarity between two individuals randomly drawn from each country.

c britannica.com/place.

d Including islands of Agalega, Rodrigues and Saint Brandon.

Like in other African countries, non-communicable diseases are increasingly being reported in this region and are the cause of high rates of mortality. Promising avenues through precision medicine can bring a radical change in the health sector with more precise diagnostics tools, better preventive care and improved prognosis. Targeted therapies adapted to particular groups of the populations are more likely to succeed than traditional treatments. The potential benefits of precision public health far outweigh the initial investments for its implementation. One key area is pharmacogenomics, where knowledge of the patient’s genetic data allows for accurate drug dosage to reduce side effects and enhance efficacy. African genomes have diverse sets of variants in their pharmacogenes that can impact on drug metabolism and efficacy (Tshabalala et al., 2019). Drug metabolizing enzymes that include the CYP family, determine the amount of drug circulating in the bloodstream. Variations in their genes influence their enzymatic activities and therefore the kinetics of drug catabolism.

Precision medicine has brought new hopes for the management of many diseases such as diabetes, cardiovascular diseases and cancer through new methods for identifying those at risk. Patients in many low-income countries often seek medical assistance when their disease state is well advanced. Recent development has culminated in the discovery of large numbers of genetic variants that contribute to disease risks (Ala-Korpela and Holmes, 2020). These are used to calculate and stratify the risks of developing a particular disease. However, it is primordial to have access to the genetic data for different populations in order to use such methods. Hence the need for large-scale sequencing efforts that would have a high return for health care improvement.

Early screening can significantly improve recovery and prognosis of cancer patients. Molecular profiling of cancer and germline testing are becoming the norms for therapeutic interventions (Birner et al., 2016). Conventional subtyping methods are based on immunochemistry and pathological evidence. They assist in treatment choices and disease management; however when combined with identification of genetic variants through sequencing, optimal treatment options can be provided. Currently many countries offer such services, which have resulted in a dramatic improvement in treatment outcomes Alongside the next generation sequencing (NGS) methods, public resources providing information on molecular profiles and cancer subtypes have amplified the accessible data that clinicians can use to make decisions on diagnosis and treatment. The American College of Medical Genetics and Genomics (ACMG) provides regular updates on guidelines for the use of NGS and interpretation of variants for clinical use (Rehder et al., 2021).

## Health and Disease

SWIO island states have reported increasing incidence of non-communicable diseases (NCDs). In Madagascar the health care system is organized into different centers at the district, regional, community and university levels namely: 1) CHRD, Centre Hospitalier de Référence de District; 2) CHRR, Centre Hospitalier de Référence Régionale; 3) CSB, Centre de Santé de Base; 4) CHU, Centre Hospitalier Universitaire. Each center records the incidences of pathologies by age groups. In 2016, the Ministry of Health reported the following cases regarding NCDs. There were 7,553 recorded cases of hypertension for individuals aged 5–24 years and around 172,293 cases for people aged 25 years and over at the CSB level (Ministry of Health, Madagascar). In CHRR/CHU settings, 2077 cases causing 55 deaths in adults were reported. In addition, diabetes is also present, being more prevalent in adults aged 25 and above at the community health centers level with 13,690 cases. At the CHRD level, diabetes is quite common from an early age with 1,621 cases for 15–24 years old and 1756 cases in adults aged 25 and over. Cardiovascular diseases are more frequent as age progresses and at the CSB level, 3,324 cases from the age of 25, amounting to a total of 3,466 cases during the year. At the level of the CHRD, the number of outpatient cases reached 3,804 of which 3,053 cases were in adults aged 25 and over.

Cancers are diagnosed at the CHRD, CHRR, and CHU. In Madagascar, several types of cancer are encountered, the most frequent being breast, cervical, bronchial and skin cancer. At the CHRR and CHU 4,118 known cases of breast cancer were reported with 1,694 deaths. Cervical cancer cases recorded were 109 cases in CHRD settings. For bronchial cancers (primary and secondary), 707 cases are recorded from children in outpatient clinic. Skin cancer is more discreet with only 55 outpatient cases and six inpatient cases at the level of the CHRR and CHU. Given the general conditions in the health services, these figures are likely to be lower than the real figures. Many rural communities are far from health centers and cannot reach the medical services.

Mauritius has a rapidly ageing population with 15% of its residents above the age of 70. This is reflected in its epidemiological distribution of diseases. Noncommunicable diseases (NCDs) account for about 84% of its total disease burden (Table.2). Death rate due to NCDs for those aged 30–70, was 411 deaths per 100,000 in 2017. Cardiovascular diseases are the main cause of death (33.2%) followed by diabetes (predominately of type 2) and cancer with 23.5 and 12.8% of total deaths, respectively, in 2016. According to the 2015 National NCD Survey, the prevalence of type 2 diabetes in the Mauritian population aged 20–74 years was 20.5%: 19.6% (Male) 21.3% (Female). The ratio of known diabetes to newly diagnosed diabetes was 2:1. Metabolic control was moderately poor in 33% (HBA1c > 9) of the diabetic population. This population group is at high risk of developing complications of diabetes, with the prevalence of pre-diabetes at 19.4%. The prevalence of hypertension was 28.4%: 27.0% for women and 30.3% for men. Only 52.6% of individuals were currently on medication and 70.6% of those continued to have elevated blood pressure (i.e., above 140/90 mmHg). There is a high prevalence level of modifiable risk factors, including overweight/obesity (54.3%); alcohol consumption (52.8%) and tobacco consumption (19.3%) and low prevalence of physical activity (23.7%).

**TABLE 2:** Prevalence of diseases.

Country	Population (thousands)	Probability of dying from any of cardiovascular disease (CVD), cancer, diabetes, chronic respiratory disease (CRD) between age 30 and 70 (%)^a^	Cancer cases per 100,000 population^b^	Diabetes Prevalence (% of population ages 20–79)^c^	CVD^d^
Comoros	888	20.6	NA	12.3	8.62%
Madagascar	28,427	26.0	NA	4.5	20%
Mauritius^e^	1,273	23.2	153.2	22.0	33%
Reunion	902	NA	213.9	NA	NA
Seychelles	99	21.1	NA	12.3	34%

a https://apps.who.int/iris/bitstream/handle/10665/342703/9789240027053-eng.pdf

b https://canceratlas.cancer.org/the-burden/sub-saharan-africa/

c United Nations, Department of Economic and Social Affairs, Population Division (2019). World Population Prospects 2019, custom data acquired via website. https://population.un.org/wpp/DataQuery/

d https://www.who.int/nmh/countries/syc_en.pdf?ua=1; https://www.who.int/nmh/countries/mus_en.pdf; https://www.who.int/nmh/countries/mdg_en.pdf?ua=1.

e Including Agalega, Rodrigues and Saint Brandon.

According to the National Cancer Registry report2018^1^, there were 2,380 new cancers in 2018: 959 males and 1,421 females. There has been a slight decrease of 3.3% from 2017. Among males, prostate cancer (n = 191, 19.9%) ranks first ahead of colorectal (n = 124, 12.9%) and lung (n = 65, 6.8%) cancers. The number of female breast cancer cases (n = 570, 40.1%) is far ahead followed by colorectal (n = 104, 7.3%), ovarian (n = 99, 7.0%) and uterus (n = 87, 6.1%) cancer. Prostate cancer (n = 99, 15.1%) remains the leading cause of cancer deaths in males, followed by lung (n = 94, 14.4%) and colorectal cancer (n = 80, 12.2%) cancers. Breast cancer (n = 173, 25.6%) is still the main cause of cancer deaths in females followed by cervix (n = 63, 9.3%) and colorectal cancer (n = 58, 8.6%).

## Precision Medicine Scope and Promises

Precision medicine (PM) requires an understanding of the interactions of genetic variability, lifestyle and environment, and the individual’s health (Ramsay, 2018). It enables timely prevention and assists health professionals to make more informed therapy decisions, thus minimizing side effects and maximizing treatment efficacy. To implement precision medicine, Desmond-Hellmann (2016) emphasized the need for more accurate detection, identification, and tracking of unique traits in subpopulations since individuals or populations are unique (Fatumo, 2020; Pereira et al., 2021). However, genetic factors associated with NCDs have been unraveled by studies targeting mostly European populations.

The last 10 years have seen significant progress in elucidating African genomes and exploring their genetic diversity and disease susceptibility (Chikowore et al., 2021). Efforts from various consortia such as H3Africa (Human Heredity Health in Africa), MADCaP (Men of African Descent Carcinoma of the Prostate) and others have fueled progress in understanding the genetic determinants of complex traits via GWAS studies in African populations. Gurdasani et al. (2019) obtained and analyzed whole-genome data from 14,126 individuals across Africa and observed statistical differences in heritability for traits between African and European populations. Their study revealed novel genetic variants associated with several traits/diseases.

Similar studies carried out by H3Africa provide a robust research and health platform for data analysts and health care professionals to generate and analyse reference datasets from control and disease cohorts (Mulder, 2017). However, participant recruitment in Africa remains a big challenge for researchers in precision medicine (Adebamowo et al., 2018) mainly due to fears and mistrust. Although progress has been made, pharmacogenomics in African populations is still an underrepresented area of research in precision medicine (Mulder, 2017). For these reasons, the African Academy of Sciences has issued a policy paper to promote the implementation of genomic medicine for public health in Africa (African Academy of Sciences, 2021).

Bentley et al. (2020) summarized the achievements of recent genomics projects in Africa. For examples, new loci, new variants within known loci, and new pharmacogenomic loci all relevant for individuals with African ancestry were discovered. However, although progress has been made, there are still many challenges the most important being the diversity of African populations and the reduced linkage disequilibrium across the genome (Choudhury et al., 2020, 2018; Fan et al. 2019). For this reason, studies of African populations require larger sample sizes in order to properly interrogate the variation present.

Though NCDs represent 84% of disease burden in Mauritius, the only actions that have been taken so far are mainly prevention through lifestyle changes. Very few studies have been done with regards to genetic screening locally. The most prevalent cancers in Mauritius are breast, prostate, colorectal and ovarian^2^. Cancer result partly from genetic components and genomics is imperative for screening, investigation, risk stratification and targeted therapeutics. The only study published in Mauritius on breast cancer genetics showed a mutation in the BRCA2 6503delTT in two sisters of the same family of Indian origin (Khittoo et al., 2001). The fact that 25% of the breast cancer identified are in women under the age of 50 points towards a strong genetic predisposition. Genomics provides crucial information towards risk-stratification of patients which has an important bearing on therapeutics. Currently, molecular profiling using limited arrays such as MammaPrint are being used to determine whether early-stage breast cancer patients need to go on chemotherapy. In Mauritius, breast cancer is typed only for estrogen receptor, progesterone receptor and HER2. Screening for BRCA1 and BRCA2 is not available in the public sector despite concomitant high incidence of ovarian cancer. One private health care center has recently advertised for cancer screening through genetic testing.

Diabetes has both genetic and environmental predisposing risk factors. Most cases of diabetes are polygenic. Genome wide association studies have identified a number of susceptibility loci for type 2 diabetes. These genetic variants have been used to develop genetic risk scores as a tool for prediction of type 2 diabetes. Monogenic forms of the disease have also been identified. These include HNF1A mutation for maturity onset diabetes of the young (MODY) and KCNJ11 mutations for neonatal diabetes. Both these conditions are highly responsive to sulfonylurea therapy. Though the incidence of MODY is only about 2%, it is critical to identify those patients in order to achieve a good glycaemic control. No genome wide study or any screening for MODY has been carried out in Mauritius so far even though 45% of the population being either diabetic or prediabetic.

Few studies on genomes from Madagascar have been carried out to investigate the population structure. Genetic exploration of the 80 or so groups from across the island have been genotyped for >2.6 m markers on an array chip (Pierron et al., 2017). Other samples for 21 Mikea (hunter-gatherers), 24 Vezo (seminomadic fishermen), and 24 Temoro (farmers) have also been studied through genotyping (Pierron et al., 2014). The African ancestry was shown to be about 67% and the main gene flow from Africa was mainland Bantu speakers. The Austronesian gene flow is about 30%. The source of the Malagasy DNA was found to originate from southern Sulawesi, the Lesser Sunda islands and eastern Borneo (Kusuma et al., 2015). The sequences of mitochondrial DNA from 257 villages across the island together with paternal Y chromosome genotypes and genome-wide SNP typing information have confirmed the Bantu and Indonesian contributions to the Malagasy genomes; while smaller components originate from European and Arab ancestries (Pierron et al., 2017).

## Conclusion and Recommendations

There is a dearth of genetic data from this part of Africa, where medical research has been mostly neglected (Rotimi, 2017; Tucci and Akey, 2019). Much of the work in Madagascar has focused on determining ancestry while none seem to have investigated disease related variants. Genomics technologies are in use but mainly for pathogens genomes at the Pasteur Institute of Madagascar and in a few laboratories in Mauritius (Table 3). This gap in scientific knowledge should be urgently addressed if the SWIO region is to benefit from the rapidly developing tools for genomic medicine. Given the complexities and costs of genomics research, one possible approach is to launch a regional initiative that would address the needs of all participating countries through a concerted effort of existing institutions (Table 3). Common training and infrastructure (eg biorepository and computing resources) can be shared. There is hope that the countries can leverage on the available resources and skills in some parts of Africa such as South Africa and West Africa where there has been considerable investment in genomics and bioinformatics through local and international efforts. The current pandemic has brought to light the unpreparedness of the scientific communities and the government bodies to address a serious public health issue. NGS platforms have made sequencing more affordable and accessible to even remote parts of Africa. Governments have to push this forward to bring the benefits of genomic medicine to the communities. It is often argued that PM is for rich countries while others will not afford it. There is however a clear economic gain in implementing PM within an electronic health care system that will allow better tracking of individuals for disease monitoring. The return on investment will be the immediate benefits to the community of innovative diagnostics and targeted treatment. Judicious use of drugs, based on pharmacogenomic evidence is far more effective and less costly than applying dosages recommended for European cohorts. There is a soaring demand for molecular profiling among families of cancer patients in Mauritius and elsewhere; most are turning to other countries to access the services. This does not guarantee return of the sequence data to the country. The scientific communities in SWIO have high hopes that molecular profiling through genomics will become the norm for health improvement. For this to happen, governments will need evidence from genetic data to assess the benefits of implementing genomics in health. Ethical issues and community engagement form an integral part of the process.

**TABLE 3:** Research institutions for genomics.

Country	Local institutions that partner with international institutions to conduct genomics research	Genome sequencing of local individuals
Comoros	Laboratoire de Biologie de l'Hôpital El-Maarouf, Moroni	Yes; https://dx.doi.org/10.1038%2Fejhg.2010.128-Y and M chromosomes
Madagascar	Université d’Antananarivo, Pasteur Institute of Madagascar	Yes; https://doi.org/10.1073/pnas.1704906114.
Mauritius	Ministry of Health and Wellness	NA
	University of Mauritius	
Reunion	CIRAD, Université de La Reunion	NA
Seychelles	NA	NA

## Data Availability

The original contributions presented in the study are included in the article/supplementary material, further inquiries can be directed to the corresponding author.

## Footnotes

1
https://health.govmu.org/Documents/Statistics/Documents/National%20Cancer%20Registry%20%20-%202018.pdf

2
https://gco.iarc.fr/today/data/factsheets/populations/480-mauritius-fact-sheets.pdf
